# A Flow Cytometric Assay to Detect Functional Ganglionic Acetylcholine Receptor Antibodies by Immunomodulation in Autoimmune Autonomic Ganglionopathy

**DOI:** 10.3389/fimmu.2021.705292

**Published:** 2021-06-23

**Authors:** Nicolás Urriola, Judith M. Spies, Katrina Blazek, Bethan Lang, Stephen Adelstein

**Affiliations:** ^1^ Department of Clinical Immunology and Allergy, Royal Prince Alfred Hospital, Sydney, NSW, Australia; ^2^ Faculty of Medicine and Health, The University of Sydney, Sydney, NSW, Australia; ^3^ Department of Neurology, Royal Prince Alfred Hospital, Sydney, NSW, Australia; ^4^ Centre for Kidney Research, Children’s Hospital at Westmead, Sydney, NSW, Australia; ^5^ Nuffield Department of Clinical Neurosciences, University of Oxford, Oxford, United Kingdom; ^6^ Central Sydney Immunopathology Laboratory, NSW Health Pathology, Sydney, NSW, Australia

**Keywords:** immunoassay, flow cytometry—methods, neuroimmunology, autoimmune autonomic ganglionopathy, diagnostic test

## Abstract

Autoimmune Autonomic Ganglionopathy (AAG) is an uncommon immune-mediated neurological disease that results in failure of autonomic function and is associated with autoantibodies directed against the ganglionic acetylcholine receptor (gnACHR). The antibodies are routinely detected by immunoprecipitation assays, such as radioimmunoassays (RIA), although these assays do not detect all patients with AAG and may yield false positive results. Autoantibodies against the gnACHR exert pathology by receptor modulation. Flow cytometric analysis is able to determine if this has occurred, in contrast to the assays in current use that rely on immunoprecipitation. Here, we describe the first high-throughput, non-radioactive flow cytometric assay to determine autoantibody mediated gnACHR immunomodulation. Previously identified gnACHR antibody seronegative and seropositive sera samples (RIA confirmed) were blinded and obtained from the Oxford Neuroimmunology group along with samples collected locally from patients with or without AAG. All samples were assessed for the ability to cause gnACHR immunomodulation utilizing the prototypical gnACHR expressing cell line, IMR-32. Decision limits were calculated from healthy controls, and Receiver Operating Characteristic (ROC) curves were constructed after unblinding all samples. One hundred and ninety serum samples were analyzed; all 182 expected negative samples (from healthy controls, autonomic disorders not thought to be AAG, other neurological disorders without autonomic dysfunction and patients with Systemic Lupus Erythematosus) were negative for immunomodulation (<18%), as were the RIA negative AAG and unconfirmed AAG samples. All RIA positive samples displayed significant immunomodulation. There were no false positive or negative samples. There was perfect qualitative concordance as compared to RIA, with an Area Under ROC of 1. Detection of Immunomodulation by flow cytometry for the identification of gnACHR autoantibodies offers excellent concordance with the gnACHR antibody RIA, and overcomes many of the shortcomings of immunoprecipitation assays by directly measuring the pathological effects of these autoantibodies at the cellular level. Further work is needed to determine the correlation between the degree of immunomodulation and disease severity.

## Introduction

Autoimmune Autonomic Ganglionopathy (AAG) is a rare disorder where autoantibodies against alpha3 containing ganglionic acetylcholine receptors (gnACHR) interrupt acetylcholine signaling in autonomic ganglia, most commonly leading to orthostatic hypotension, gastrointestinal dysmotility, degrees of anhidrosis (1, 2), and less commonly, abnormal or absent pupillary light reflexes, dry eyes and mouth, as well as dysfunction of genitourinary, cardiovagal and erectile systems (3).

**Figure 1:**
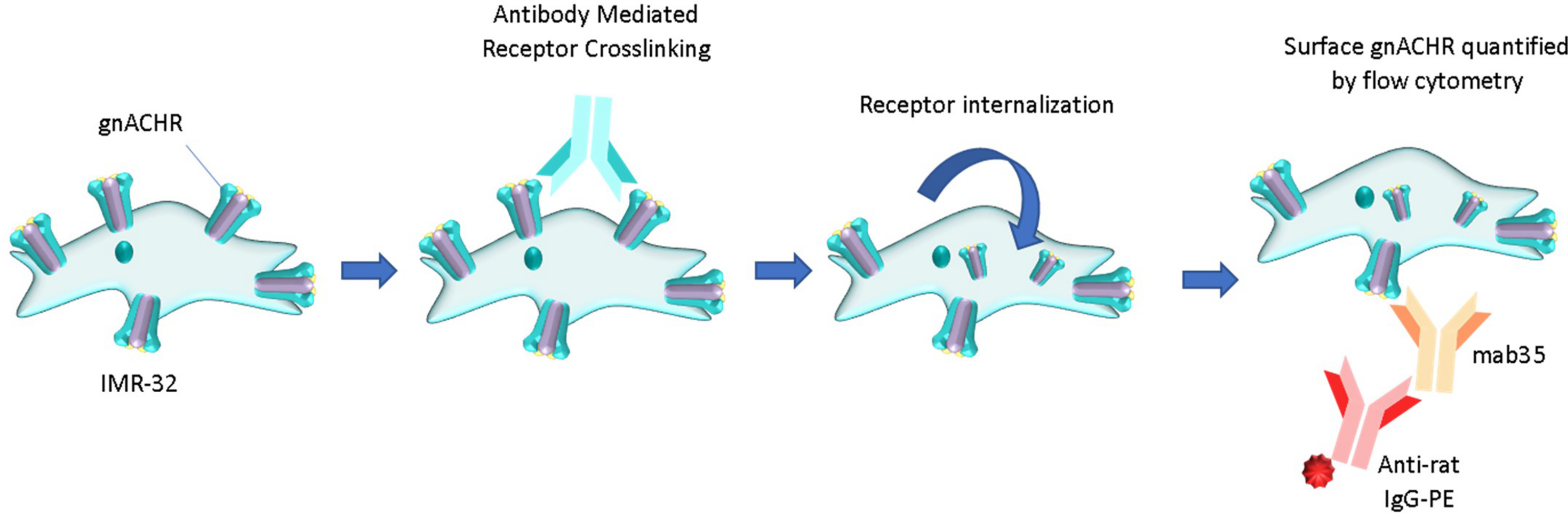
Pathology of autoantibody-induced cell surface receptor crosslinking and internalization (immunomodulation). The remaining surface receptors can be comparatively enumerated flow cytometrically. PE, phycoerythrin.

The gnACHR are present on the surface of neurones. Autoantibodies in immune-mediated neurological diseases that target plasma membrane surface receptors have the potential to produce pathology by one or more of three mechanisms; *binding* to the target (thus enabling secondary toxicity *via* complement deposition, or mediation of Antibody Dependent Cellular Cytotoxicity), *blocking* interaction with the receptor’s native ligand, or *immunomodulation*, whereby two receptors are cross-linked, causing receptor internalization and effective removal from the plasma membrane (4) (**Figure 1**). Assays for the detection of antibodies binding to (3, 5) and blocking (3) gnACHR have been described; of note, blocking antibodies are not seen without accompanying binding antibodies. Research assays thus far implicate that the universal pathophysiology of autoantibodies that target the gnACHR in AAG is through receptor immunomodulation, as shown by functional electrophysiological (patch-clamp) studies (6) and confocal immunofluorescence imaging (7).

Binding assays include the radioimmunoprecipitation assay (RIA) and the Luciferase Immunoprecipitation assay (LIPS), that are reported to have a sensitivity and specificity of 50 and 100%, respectively (3, 5), with antibody levels directly correlated with severity of autonomic impairment. The RIA is, however, only moderately specific (92%) in AAG with severe autonomic dysfunction, with low positive levels not associated with autonomic impairment (8). Likewise, positive results can be seen in up to 16.3% of patients with autoimmune disorders without autonomic impairment when assessed by the LIPS assay (9).

Previous studies have shown that muscle-type ACHR receptor-modulation can be detected reliably in a semi-automated fashion and free of the use of radioligands by flow cytometry in the diagnosis of myasthenia gravis (10). Here, we show that this assay can be adapted for the diagnosis of AAG and that the results correlate completely with the RIA.

## Materials and Methods

### Serum Samples

AAG was defined as an immune-mediated autonomic failure syndrome, without evidence of motor or sensory nerve deficits. Seropositive or negative AAG samples were defined by the presence or absence of gnACHR antibodies as established by RIA, respectively.

One hundred and ninety samples were assessed for gnACHR immunomodulation: Plasma and serum from one patient with seropositive AAG (established by RIA) was used to validate the novel flow cytometric assay, and this was then validated with two further seropositive samples obtained from the Nuffield Department of Clinical Neurosciences, Oxford. Nine other samples were also supplied by the Oxford group; a mixture of seropositive [full clinical characterization of these and other samples has recently been published by Koay et al. (11)] and seronegative samples that were blinded for gnACHR antibody status. RIA was performed as previously described, in brief, solubilized IMR-32 membranes containing gnACHR were complexed with ^125^I-epibatidine prior to the addition of serum, then immunocomplexes were precipitated out. Positive samples (>100pM of ^125^I-epibatidine bound complexes) were serially diluted with results expressed as pM of epibatidine precipitated per liter of serum (3).

177 samples were obtained locally, and clinically categorized before performance of the flow cytometric-based immunomodulation assessment as; probable AAG not confirmed serologically (i.e. clinically diagnosed with features consistent with AAG without formal gnACHR autoantibody assessment *via* RIA, 1 sample), “Seronegative AAG” (i.e. patients whose clinical features were consistent with AAG but who had negative gnACHR antibodies by RIA; 5 samples), Healthy Controls (39 samples), Autonomic Disorders not thought to be AAG (43 samples), Other Neurological Disorders (47 samples) and patients diagnosed with Systemic Lupus Erythematosus without autonomic impairment (SLE – 42 samples; **Tables 1**–**3**).

**Table 1:** Clinical diagnoses of samples tested for gnACHR immunomodulation.

Disease category	No. of samples
Seropositive AAG	4
Blinded Oxford samples	9
Probable AAG	1
Seronegative AAG	5
Healthy Controls	39
Autonomic Disorders (not AAG)	43
Other Neurological Disorders	47
SLE	42
**Total**	**190**

Blinded Oxford Samples consisted of specimens that were either positive or negative for gnACHR antibodies by RIA. AAG, Autoimmune Autonomic Ganglionopathy; SLE, Systemic Lupus Erythematosus; (as identified by the Systemic Lupus International Collaborating Clinics (SLICC) criteria).

**Table 2:** Clinical diagnoses of samples from subjects with a range of disorders with autonomic dysfunction, but not thought to be autoimmune autonomic ganglionopathy.

Autonomic Disorders (non-immune)	No. of samples
POTS	9
Isolated GIT dysmotility disorder	6
Isolated orthostatic hypotension	5
PAF	4
Autonomic Neuropathy due to DM	3
Autonomic Neuropathy due to surgery/radiotherapy	3
Isolated hypohidrosis/anhidrosis	3
Autonomic and large fiber sensory neuropathy	2
Autonomic Neuropathy due to renal failure	2
Autonomic neuropathy due to vasculitis	1
GIT dysmotility due to (bulky) tumor	1
Orthostatic hypotension due to Congestive Cardiac Failure	1
Orthostatic hypotension due to deconditioning	1
Chronic Constipation	1
MSA with severe autonomic dysfunction	1
**Total**	**43**

POTS, Postural Orthostatic Tachycardia Syndrome; PAF, Pure Autonomic Failure; MSA, Multiple System Atrophy.

**Table 3:** Clinical diagnoses of samples from subjects with a range of neurological diagnoses without autonomic dysfunction.

Other Neurological Diseases	No. of samples
Multiple Sclerosis	10
Myasthenia gravis (mnACHR-ab positive)	6
NMOSD (AQP-4-ab positive)	4
PCD (seronegative)	4
CIDP	2
MND (ALS)	2
Paraneoplastic sensory neuropathy	2
Parkinson’s Disease	2
Seizure disorder	2
Stiffperson Syndrome (GAD-ab positive)	1
NMDAR-ab encephalitis	1
AMPAR-ab encephalitis	1
GlyR-ab encephalitis (PERM)	1
Morvan’s Syndrome (seronegative)	1
NMOSD (AQP-4-ab negative)	1
Viral meningitis (PCR negative)	1
Optic Neuritis (AQP-4-ab/MOG-ab negative)	1
Seronegative autoimmune encephalitis	1
Central canal stenosis	1
Creutzfeldt-Jakob Disease	1
Immune-mediated hearing loss	1
Sensory Ganglionopathy	1
**Total**	**47**

PCD, Paraneoplastic Cerebellar Degeneration, as diagnosed with rapidly progressive clinical evidence of cerebellar disease with or without cerebellar atrophy on imaging; malignancies were histologically confirmed as metastatic squamous cell carcinoma (1 patient), lung adenocarcinoma (2 patients) and breast cancer (1 patient). Multiple Sclerosis as defined by the McDonald criteria, 2010. mnACHR-ab, muscle-type nicotinic acetylcholine receptor antibody; NMOSD, Neuromyelitis Optica Spectrum Disorder; AQP-4-ab, Aquaporin-4 antibody; CIDP, Chronic Inflammatory Demyelinating Polyradiculopathy, as per diagnostic guidelines set forth by the European Federation of Neurological Societies and the Peripheral Nerve Society, 2010; MND, Motor Neuron Disease, as diagnosed according to the World Federation of Neurology diagnostic criteria for amyotrophic lateral sclerosis (ALS). Parkinson’s Disease diagnosis reached as per the International Parkinson and Movement Disorder Society Parkinson Disease (MDS-PD) criteria. GAD-ab, Glutamic Acid Decarboxylase antibody; NMDAR-ab, N-methyl-D-aspartate receptor antibody; AMPAR, α-amino-3-hydroxy-5-methyl-4-isoxazolepropionic acid receptor antibody; GlyR-ab, Glycine receptor antibody; PERM, Progressive Encephalomyelitis with Rigidity and Myoclonus. Morvan’s Syndrome was diagnosed clinically in combination with suggestive electrodiagnostic studies. Viral meningitis diagnosed clinically as an acute meningitis with cerebrospinal fluid pleocytosis in the absence of bacterial or fungal growth despite negative viral polymerase chain reaction (PCR) nucleic acid testing. MOG-ab, Myelin Oligodendrocyte Glycoprotein antibody.

Human Research Ethics approval was obtained from the Sydney Local Health District Ethics Review Committee, under protocol X13-0152. Serum or Plasma Exchange (PLEX) fluid was obtained after written informed consent was granted from subjects. Local patient samples were obtained between 2015-2016 from in- or outpatient services attached to the Royal Prince Alfred hospital, Sydney, on a convenience basis. Collection of samples submitted from Oxford occurred between 2005 – 2019 as previously described (11).

### Cell Culture

The neuroblastoma cell line, IMR-32, has previously been validated as a rich source of surface-expressed, fully formed, conformationally correct gnACHR (12). IMR-32 was purchased from CellBank Australia, and was grown in 25cm^2^ canted neck cell culture flasks in Eagle’s Minimum Essential Media (EMEM) + 2nM Glutathione + 1% Non-Essential Amino Acids + antibiotics (penicillin/streptomycin), supplemented with 10% Fetal Calf Serum (FCS). Cells were cultured at 37°C, 5% CO_2_ and at 95% humidity.

Media was replenished every 48-72 hours, with cells either being harvested or split when approximating 80% confluence.

### Ganglionic (alpha3) Immunomodulation Assay

For cell harvesting, media was decanted and 1mL of trypsin 0.25% (Sigma) was applied to remove the cellular monolayer from the flask surface. After inactivating the trypsin with the addition of 3mL of RPMI with 10% FCS, the cells were transferred to a sterile container, and were gently dissociated by pipetting. Cells were centrifuged, then resuspended in 1mL of fresh RPMI (without serum), with viable cells enumerated with a hemocytometer and trypan blue exclusion. The concentration was adjusted so that 190µL contained 2x10^5^ cells. 190µL aliquots were then transferred to a sterile 96-well flat-bottom tissue culture plate and left to incubate overnight to allow re-expression of gnACHR affected by trypsin-mediated degradation.

The following day, 10 µl of serum for analysis was added to individual wells (1:20 dilution), and 10µl of FCS to two wells to serve as controls for background/autofluorescence (unstained cells) and maximally stained cells. The plate was then incubated overnight (12-15 hours) at 37°C, 5% CO_2_ and at 95% humidity.

The supernatant was then gently decanted and 100µL of wash buffer (calcium/magnesium-free Hanks Balanced Salt Solution + 1% FCS + 0.1%NaN_3_) containing Fixable Viability Stain 660 (FVS660, Becton Dickson) was added to each well, then cells were removed with gentle pipetting and transferred to a V-bottom 96-well microtiter plate, and allowed to incubate for 10 minutes at room temperature. Cells were washed twice with 200µL of wash buffer, with centrifugation set at 645g, and excess wash solution removed by blotting the plate on absorbent paper.

Following this, cells were fixed with 100µL of 1% paraformaldehyde in wash buffer at 4°C for 15 minutes, then washed a further two times by centrifugation at 880g prior to blocking with 100µL of 10% FCS in wash buffer at room temperature for 30 minutes on gentle rotation. Cells were centrifuged again, and resuspended with 100µL of primary antibody (mab35- Sigma) at 2µg/mL. Unstained cells were incubated in wash buffer without primary antibody. The plate was left to incubate overnight in the dark at 4°C.

Cells were washed a further two times, then stained with 100µL of a 1:1000 dilution of a secondary antibody (anti-rat IgG, conjugated to phycoerythrin, Southern Biotech), and left to incubate at room temperature for 30 minutes on gentle rotation. Cells were washed twice more, then resuspended in 1% paraformaldehyde to a final volume of 200µL until analysis (either immediately, or the next day).

### Flow Cytometric Evaluation of Surface alpha3-Containing gnACHRs

**Figure 2:**
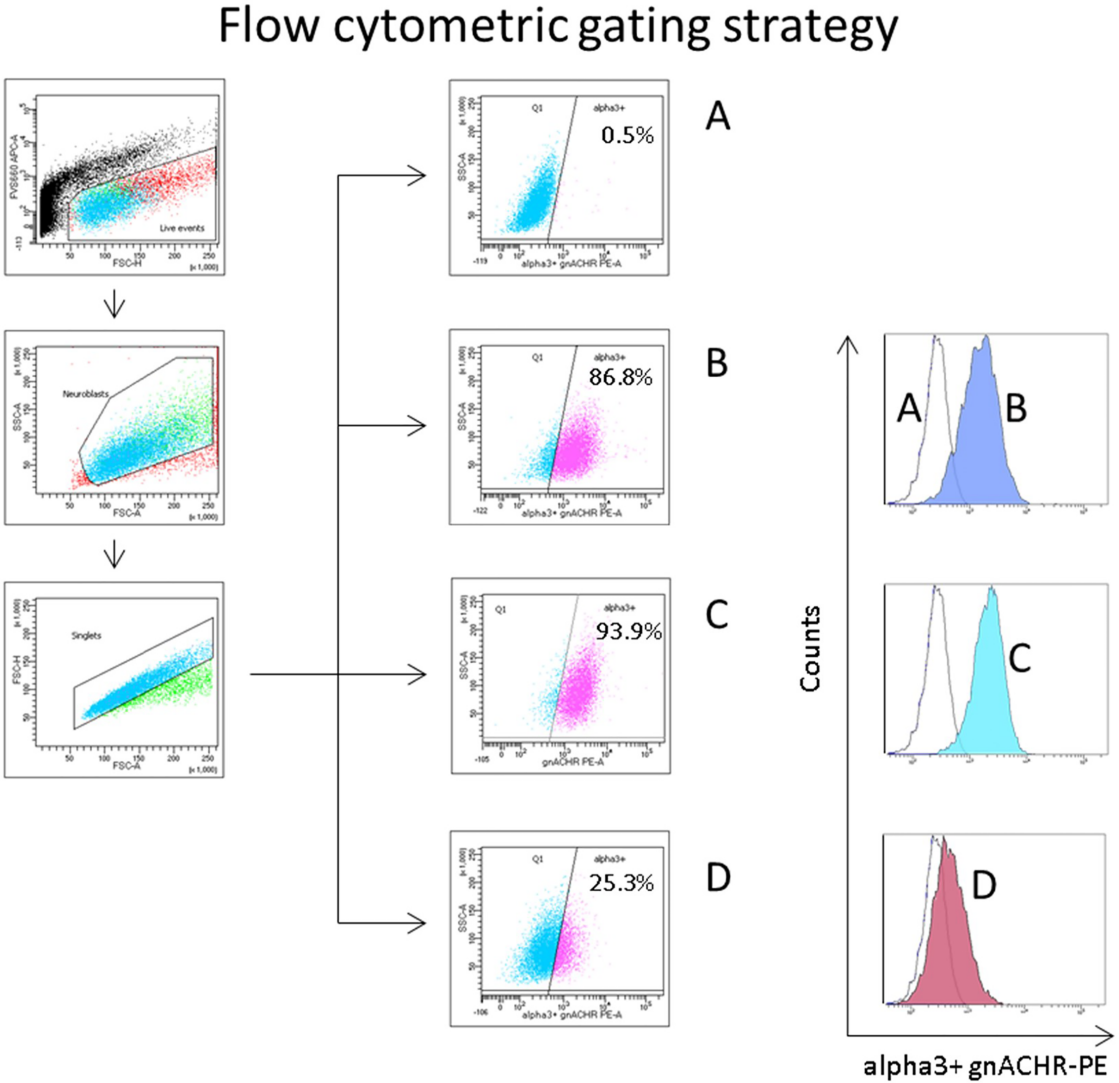
Flow cytometric gating strategy to quantify autoantibody induced ganglionic acetylcholine receptor (gnACHR) internalization. Cells are gated on live events (FVS660-low, through the APC channel), then subgated on small ‘neuroblast’ events with doublets excluded. The amount of gnACHR remaining on the surface of cells after sample serum is added is quantified by the amount of mab35 (followed by a PE-conjugated anti-rat IgG antibody) staining evident. (Sample **A**) incubated with Fetal Calf Serum (FCS), and only stained with a secondary antibody (unstained cells). Flow plots (middle of figure) display the percent of events positive for gnACHR as compared to the unstained cells. Histograms (to the right) display the same data. (Samples **B–D**) incubated with serum and stained with both primary and secondary antibodies. **(B)** = FCS (maximally stained cells), **(C)** = (typical) healthy control serum, **(D)** = serum from a patient with confirmed seropositive Autoimmune Autonomic Ganglionopathy.

Cells were transferred to FACS tubes and analyzed on a BD FACSCANTO II. Gating was set to exclude non-viable events (prior to fixation) based on low FVS660 (APC channel) fluorescence, with subgating set around the discrete cell population evident on a forward and side scatter plot. Approximately 10,000 events of interest were counted in each test. This population was interrogated on a final flow plot (SSC-a *vs* PE), with a quadrant gate based on the unstained stained cell sample such that only 0.5-1% of events were ‘positive’ for alpha3 containing gnACHRs (**Figure 2**).

### Data Analysis

The amount of receptor immunomodulation was calculated as described by *Lozier* et al (10), although the proportion of gnACHR positive events based on gating was utilized, according to the formula:

$$\%\;Immunomodulation=\left[1-\left(\frac{\text{Test Sample}-\text{MIN}}{\text{MAX}-\text{MIN}}\right)\right]\times 100$$

where ‘Test Sample’, ‘MIN’ and ‘MAX’ refer to the proportion of (gated) events positive for gnACHR in the tested sample and controls (unstained and maximally stained cells), respectively.

After testing was complete, the samples from Oxford were unblinded for the results determined by RIA and analyzed in an “intention-to-test” fashion (13); all seropositive (by RIA) samples were included with the known positive controls in a final group – “seropositive AAG”, while the remainder were pooled with the data generated from the Healthy Controls (n =39 + 5 = 44). The mean of this Healthy Control group was calculated, with the decision limit for positivity set at greater than the mean and three standard deviations (mean + 3SDs). This decision limit was then applied to the remaining samples.

A Receiver-Operator Characteristic (ROC) curve was constructed, comparing the percent immunomodulation of the seropositive AAG samples, with that from the Healthy Control, Autonomic Disorders (not thought to be AAG), Other Neurological Disorders and SLE groups cumulatively.

Endpoint titers of positive results were established by two-fold dilutions to ascertain the titer prior to no significant immunomodulation being observed (i.e., less than the decision limit). Immunomodulation results (percent of events gated at the initial dilution and endpoint titer) were each compared to RIA results (pM). Spearman’s rank correlation rho was calculated due to the relationship being non-linear monotonic. Analyses were conducted with R, version 4.0.2 (14) and the tidyverse suit of packages (15). ROC curves were generated with ROCit (16).

## Results

**Figure 3:**
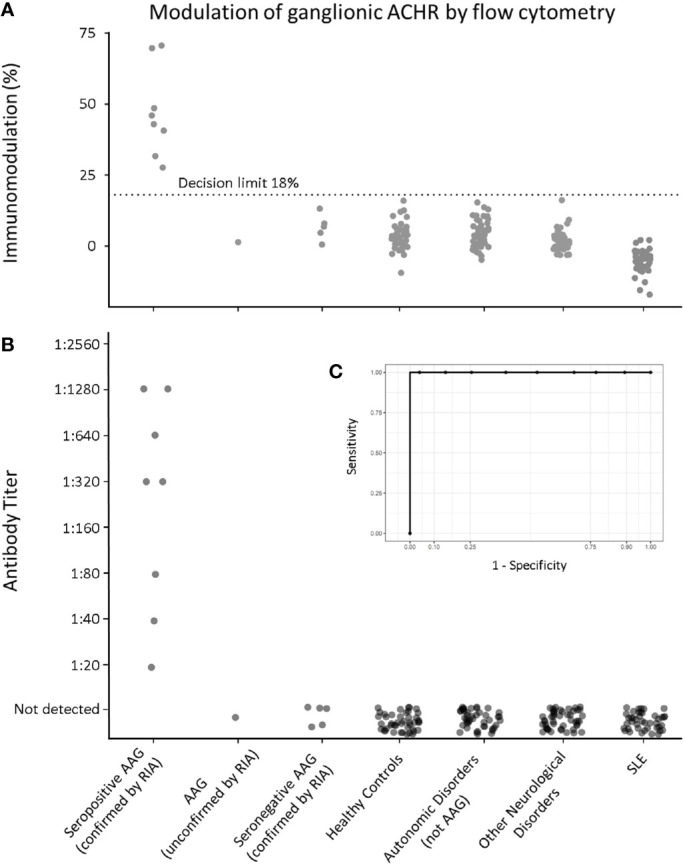
Flow cytometric gnACHR modulation by patient autoantibodies. **(A)** Immunomodulation at screening dilution of 1:20. **(B)** Endpoint Titer of results (results with <18% immunomodulation being considered “Not Detected”). (**C** – inset) Receiver-Operator Characteristic (ROC) curve for all radioimmunoassay confirmed seropositive AAG serum samples, compared to all expected negative samples (Healthy Controls, Autonomic Disorders, Other Neurological Disorders and Systemic Lupus Erythematosus). Aurea Under ROC (AUROC) = 1).

Sera from all patients with AAG, including the samples later shown to have been positive when initially assayed by RIA at Oxford, yielded immunomodulation levels greater than the threshold value of 18% (the mean immunomodulation of the Healthy Control group (3.45%) plus three standard deviations). **Figure 3A** demonstrates that no samples from any of the control groups displayed a signal above 18%.

Importantly, there was complete qualitative congruence between results obtained by RIA and the novel flow cytometric assay; all positive samples by RIA were positive by flow cytometry, and similarly, all samples from disease and Healthy Controls were negative by flow cytometry.

Utilizing this immunomodulation decision limit (18%), none of the patients in the ‘seronegative AAG’ group were positive, and neither was the one local patient with probable AAG (but who had not had RIA testing). **Figure 3B** shows the endpoint titer determination of all samples, with results having less than 18% modulation being designated as ‘Not Detected’.

A ROC curve comparing all the RIA-confirmed seropositive group (8 samples) with the pooled, expected negative samples (Healthy Controls – including negative samples from Oxford that were initially blinded, Autonomic Disorders not thought to be AAG, Other Neurological Disorders and SLE samples; total 169 samples) showed excellent discrimination at 18% modulation, with an area under the ROC (AUROC) of 1, yielding 100% sensitivity and specificity for serostatus (**Figure 3C**).

**Figure 4:**
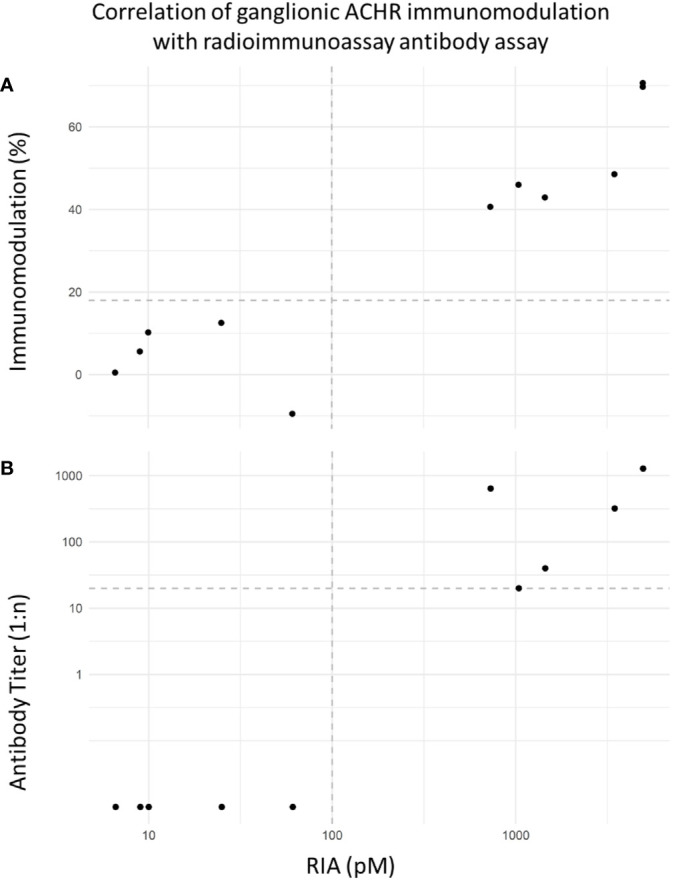
**(A)** Correlation of flow cytometric-based immunomodulation assay (1:20 screening dilution) with radioimmunoprecipitation assay (RIA). Decision limits set at 18% immunomodulation and 100pM respectively. Correlation (Spearman r = 0.897). The RIA values from the seropositive AAG samples from Oxford ranged from 730-3464pM (RI < 100). **(B)** Correlation of flow cytometric immunomodulation endpoint titer results (decision limit set at positivity starting at 1:20 dilution) and RIA. Correlation (Spearman r = 0.896).

**Figure 4** demonstrates the correlation between gnACHR immunomodulation detected by the novel flow cytometric assay described here and expressed as either the extent of immunomodulation at the initial dilution, or the endpoint titer, and the amount of binding detected by RIA performed at University of Oxford; both flow cytometry based results correlated well with the RIA values (r = 0.897 and 0.896 with percent and endpoint titer immunomodulation determinations, respectively).

## Discussion

This study demonstrates that immunomodulation of gnACHR on IMR-32 cells as determined by flow cytometry performs at least as well as RIA in the detection of pathogenic gnACHR antibodies (**Figures 3**, **4**). In AAG with pathogenic gnACHR antibodies, the autoantibodies exert their effects by immunomodulation; other mechanisms for disease causation have not been consistently demonstrated. The assay described in this paper utilizes this function of gnACHR antibodies in establishing a diagnostic assay that therefore minimizes the possibility of ‘false-positive’ results (6). Other available assays such as RIA (3) and LIPS (5) rely on immunoprecipitation of the gnACHR or its subunits and therefore do not give a functional readout of the autoantibody pathogenicity. These assays may also detect intracellular portions of the receptor that in turn may lead to false positive results. For example, we found no positivity in a sizeable SLE cohort or in heterogenous groups of non-AAG neurological disorders (**Figure 3**) contrary to other reports (9). The absence of positive results in patients without autonomic failure in our study is consistent with findings in other assays that are dependent on detection of plasma surface antibody-mediated receptor internalization, for example glycine-receptor antibodies (17). We found a complete qualitative concordance with RIA seropositive samples, the method that has to date been considered the gold standard for detection of these antibodies. None of the seronegative (by RIA) AAG patients were positive by the immunomodulation assay, although further work in a larger population of RIA-negative AAG patients would be needed to confirm that low affinity (but functionally relevant) autoantibodies are indeed absent.

**Figure 4** shows excellent separation between positive and negative samples and a good correlation between the two methods of quantitation of receptor internalization and the gnACHR RIA result. These two measures of the degree of immunomodulation were, however, not mutually predictable. For example, one seropositive sample displayed 46% immunomodulation at the initial dilution of 1:20, but did not exert significant receptor internalization at higher titers; another positive sample had a lower initial immunomodulation of 40.6%, but still exhibited detectable receptor modulation at a 1:640 dilution. Further work is needed to establish if there is a correlation between disease severity and the extent of immunomodulation at the initial dilution of sera or the endpoint titer. Currently, negative results are expressed as <18% immunomodulation at a 1:20 serum dilution, with positive results (greater than 18% immunomodulation) being subjected to additional serial titration.

Mab35 has previously been previously validated for correctly identifying α3 containing ACHRs on the surface of IMR-32 (18). Mab35 binds only to fully assembled acetylcholine receptors (19), eliminating unwanted binding to partially assembled subunits. Other commercially available antibodies and fluorescent probes purported to bind to gnACHRs were trialed, but none served adequately for this assay (data not presented; manuscript in preparation). It was therefore not possible to determine analytical sensitivity of the assay. However, the finding that a sample with a gnACHR antibody level of 730pM continued to exert significant receptor internalization despite a further 32-fold dilution to an equivalent calculated concentration of 23pM of gnACHR antibodies implies excellent analytical sensitivity (data not shown).

IMR-32 was selected as the cell line for this assay due to its constitutive high expression of gnACHR (12). Although the dominant gnACHR consists of α3β4 subunits (12), autoantibodies in AAG recognize a diverse range of α3-containing subunits; while transiently transfected cell lines have increasingly become an attractive option to study antibody mediated neurological disease, cell lines dually-transfected with α3 and β4 subunits have fewer binding sites for patient-derived gnACHR autoantibodies than native IMR-32 despite α3β4 gnACHR assembly (20), probably due to the expression of gnACHR containing α5 or β2 subunits with α3 subunits in addition to the α3β4 receptors found in the IMR-32 cells. Furthermore, unlike muscle ACHR, the mechanism of trafficking of gnACHR to the plasma membrane surface, associated scaffolding proteins and clustering mechanisms are not well elucidated and as such are difficult to replicate outside of their native neuronal environment (21).

IMR-32 was, however, a challenging cell line to adapt to a flow cytometric immunomodulation assay. IMR-32, like many neuroblastoma cell lines, is weakly adherent. Initial attempts to perform all wash steps in the flat-bottom cell culture plate by leaving the cell monolayer intact were unsuccessful, as even gentle washing would dissociate entire sheets of cells. Furthermore, IMR-32 is known to produce a thick acellular proteinaceous substance after several days of culture (22), and, additionally, we found that, IMR-32 displayed/underwent a high-degree of anoikis. Together, these factors caused difficulty isolating a consistent population by forward and side scatter, as significant amounts of debris and non-viable cells would overlie the viable cell population (data not shown). However, IMR-32 could be forced to perform well for our purposes by seeding a large number of cells onto the culture plates with an overall 48-hour incubation period for growth so as to allow near confluence in order to minimize anoikis. Viability staining and multiple washes allowed further delineation of the cell population of interest. During the development of this assay, considerable difficulty arose when attempting to stain and counterstain unfixed cells, due to a combination of receptor internalization by mab35 itself (a potent gnACHR immunomodulator (23)), and rapid cellular deterioration by the end of the staining procedure. This necessitated early fixation of cells during the staining protocol. Fortunately, mab35 is able to bind to paraformaldehyde fixed targets, overcoming these difficulties.

Finally, by utilizing a cell line that constitutively produces the receptor of interest (gnACHR), stocks of the antigen of interest can be guaranteed as the cell line is immortal. This circumvents the need for solubilized receptor (such as that used for the RIA), traditionally employing sacrificial athymic mice to grow gnACHR expressing xenotumours (3, 18).

The flow cytometric assay we have developed has the following advantages; firstly, an immunomodulation assay demonstrates autoantibody pathological functionality at the cellular level. Secondly, the radioligand needed for the RIA (^125^IPH, commonly referred to as radioionidated epibatidine), makes cost a significant barrier to gnACHR binding assay establishment, as the initial high cost of the product can only be offset by a high volume of test requests before radioactive decay renders the ligand unusable (approximately 8 weeks) – which for a rare disease may be unworkable in countries with smaller populations. Radioactive ligands also pose safety concerns in the modern laboratory. Furthermore, we found ^125^IPH to be unstable at ambient (or higher) temperatures, making this ligand unusable in the potential immunomodulation radioimmunoassays that are dependent on prolonged incubations at 37°C.

Another assay that has been used to detect gnACHR is the LIPS assay (5) that is dependent on transient transfection of separate ganglionic receptor subunits, which, therefore, impedes the capacity of the assay to detect autoantibodies that recognize only fully formed receptors. Furthermore, an irrelevant antibody may potentially recognize recombinant subunits but not completed receptors; this may affect the sensitivity and specificity of the assay (24). Additionally, luciferase-based assays are exquisitely sensitive and may be prone to a high degree of false positivity, particularly with ‘sticky’ serum displaying increased non-specific binding, such as from patients with inflammatory disorders. These factors may explain the unexpected findings discussed above of a high proportion of gnACHR subunit specific antibody positivity in patients with SLE assessed by LIPS (9.4% (9)), even without autonomic dysfunction., which was not seen in our immunomodulation assay. The RIA assay also displays great analytical sensitivity, but at the cost of specificity when the decision limit is low; the current decision limit for the gnACHR antibody RIA performed at Mayo Clinic (Rochester) is 20pM (25), although this test only starts being reasonably specific for clinically meaningful autonomic failure at 200pM, and very specific at levels above 400pM (8), which is further verified in a study by the Oxford group in which AAG patients, when seropositive for gnACHR antibodies, have levels that exceed 200pM (11).

An overly generous decision limit of 20pM can lead to a false positivity rate of up to 86% for gnACHR antibody testing in real-world settings, potentially leading to unnecessary reflex investigation and intervention (26). Of interest, the initially reported (and often repeated) sensitivity and specificity of the RIA in (non-tumor associated) AAG was said to be 50 and 100%, respectively, based on a decision limit of 50pM (3). Applying the stricter decision limit of 200pM to the data set from this initial study would have reduced the reported clinical sensitivity from this cohort to approximately 32%, while a decision limit of 400pM would give only a sensitivity of 21%. It would be interesting to ascertain the true clinical sensitivity of functionally relevant gnACHR antibodies by immunomodulation, as the RIA would not be able to discriminate between pathological gnACHR antibodies (directed to the extracellular domain) and likely non-pathological antibodies directed against cytoplasmic determinants, as discussed above and has been determined in other neuroimmunological conditions (27).

Our study had several limitations. Firstly, we cannot disprove that another autoantibody to an as yet unidentified receptor on the surface of IMR-32 may cause inadvertent internalization of gnACHR, although we found no evidence to support this, as all RIA-proven seropositive and seronegative AAG samples gave congruent gnACHR immunomodulation results. If this phenomenon were to occur, a similar clinical phenotype would be expected secondary to gnACHR downregulation, but with discordant assay results (a negative gnACHR-antibody result by RIA and a positive result by immunomodulation). Such a scenario is not without precedent, as antibodies against MuSK can reduce muscle ACHR density from the surface of striated muscle (28, 29), and in fact would be a welcome occurrence in order to spur research in order to identify putative autoantibody targets in patients with acquired autonomic failure. The converse scenario is also theoretically possible, where a serum sample may have a high degree of gnACHR binding as measured by RIA, but without significant gnACHR immunomodulation. Presuming this scenario does exist, and the patient carries a definitive clinical diagnosis of AAG, separate experiments would be needed to exclude causes of false RIA binding (such as non-specific or specific binding to the radioligand as opposed to the target receptor, as is known to occasionally occur in other radioligand-labeled neuronal receptor RIA assays (27)), or to provide another conclusive mechanism of pathology caused by binding of these autoantibodies to the receptor in the absence of receptor internalization.

Another limitation of this study is the small sample size of positive results. AAG is a supremely rare disease with an unknown incidence, and is difficult to diagnose. To prevent data contamination by selection bias (i.e., identifying presumed AAG patients solely by our novel assay in order to circuitously prove our assay functions as intended), we obtained blinded samples from Oxford. The positive samples were recruited from a clinically well-characterized published cohort of AAG patients (11). Many of the patients were historical, without stored serum for analysis. Still, the work presented here is the largest direct comparison of gnACHR-antibody assays to date.

Regardless of these limitations, flow cytometric-based immunomodulation to gnACHR is a viable assay format for the diagnosis of AAG, and can be directly incorporated into a routine clinical diagnostic setting, without the need for specialized transfected cell lines, animal housing or radioactive material, whilst able to detect the pathology induced by gnACHR autoantibodies at the cellular level. In time, this assay may prove to be the ‘Gold Standard’ format for diagnosis of seropositive AAG, as it may avoid many of the pitfalls that cause false positive results in other assays.

## Data Availability Statement

The original contributions presented in the study are included in the article/supplementary material. Further inquiries can be directed to the corresponding author.

## Ethics Statement

The studies involving human participants were reviewed and approved by The Sydney Local Health District Ethics Review Committee under protocol X13-0152. The patients/participants provided their written informed consent to participate in this study.

## Author Contributions

Study design, implementation, and manuscript drafting by NU. Statistical analyses and their description by KB. Sample acquisition and interpretation of relevant neurological findings by JMS. Radioimmunoprecipitation studies and further sample acquisition by BL. Study oversight and contributions to manuscript by JS and SA. All authors contributed to the article and approved the submitted version.

## Conflict of Interest

The authors declare that the research was conducted in the absence of any commercial or financial relationships that could be construed as a potential conflict of interest.
